# Frequency of cardiovascular diseases in patients of psoriasis presenting at a tertiary care hospital in Karachi Pakistan

**DOI:** 10.12669/pjms.42.3.12295

**Published:** 2026-03

**Authors:** Peryanka Jagdeesh Kumar, Saadia Tabassum, Sadia Masood, Shaheen Naveed

**Affiliations:** 1 Peryanka Jagdeesh Kumar, Section of Dermatology, Department of Medicine, Aga Khan University Hospital, Karachi, Pakistan; 2 Saadia Tabassum, Section of Dermatology, Department of Medicine, Aga Khan University Hospital, Karachi, Pakistan; 3 Sadia Masood, Section of Dermatology, Department of Medicine, Aga Khan University Hospital, Karachi, Pakistan; 4 Shaheen Naveed, Section of Dermatology, Department of Medicine, Aga Khan University Hospital, Karachi, Pakistan

**Keywords:** Cardiovascular diseases, Hypertension, Psoriasis

## Abstract

**Objective:**

To determine the prevalence of cardiovascular disorders among psoriasis patients who present at a Pakistani tertiary care facility.

**Methods:**

From February 9, 2022 to August 8, 2022 a cross-sectional study was carried out in the outpatient’s Department of Dermatology, AKUH, Karachi, Pakistan. The study covered all patients who visited AKUH, Karachi and met the inclusion criteria. Following an explanation of the study’s methodology, risks, and advantages, informed consent was obtained. The proforma was filled out with all the relevant information gathered.

**Results:**

Seventy seven (48.7%) participants in our study were male, and 81 (51.3%) were female. Their ages ranged from 18 to 80 years, with a median age of 40.00. Amongst 78 patients (49.4%), cardiovascular disease was identified. The distribution of cardiovascular disease categories identified that 76 patients (97.4%) had hypertension.

**Conclusion:**

Cardiovascular disease was frequently seen in our study, with a notably high percentage of hypertension. It is advised that more extensive research with case control and bigger cohorts be done to validate the current study findings.

## INTRODUCTION

Psoriasis, a chronic inflammatory skin disorder, characterized by well-defined, erythematous plaques with silvery scales is frequently seen on the scalp and extensor surfaces.^1^ About 2-3% of people worldwide are affected with this prevalent ailment, with annual incidence rates in adults ranging from 0.08% to 0.23%.^2^,^3^ The WHO has classified psoriasis as a major non-communicable disease due to its significant impact on patients’ quality of life and the resulting physical and psychological effects.^4^ The condition ranges in severity, with moderate-to-severe forms posing greater challenges to overall health and management.^5^

Psoriasis is linked to a number of systemic comorbidities in addition to its cutaneous manifestations. Between 6% and 42% of people with psoriasis have psoriatic arthritis (PsA), Furthermore, individuals with psoriasis have a higher likelihood of developing metabolic syndrome than the general population, which is defined by diseases like obesity, diabetes mellitus, dyslipidemia, and hypertension to name a few.^6^

There is growing evidence that psoriasis considerably raises the risk of cardiovascular diseases (CVDs). According to a meta-analysis of observational studies, people with psoriasis are 1.58 times more likely than people without the condition to develop hypertension, and the risk is further elevated in severe forms of the condition.^7^ Psoriasis-related chronic inflammation raises the risk of cardiovascular disease by causing endothelial dysfunction, vascular damage, and accelerated atherosclerosis.^8^

Psoriasis is now increasingly recognizing as a systemic immune-mediated inflammatory disorder rather than a disease that is confined only to the skin.^3^ Persistent activation of dendritic cells and T-helper (Th1 and Th17) lymphocytes result in sustained release of pro-inflammatory cytokines including tumor necrosis factor-α (TNF-α), interleukin-6 (IL-6), interleukin-17 (IL-17), and interleukin-23 (IL-23).^8^ These mediators promote oxidative stress, endothelial dysfunction, and increase expression of vascular adhesion molecules, which facilitate leukocyte infiltration into the vascular intima and accelerates atherogenesis. Chronic systemic inflammation also contributes to plaque formation, instability, and thrombosis within coronary arteries, therefore increasing the risk of myocardial ischemia and other major adverse cardiovascular event. This shared inflammatory pathway between psoriasis and atherosclerosis provide a plausible biological explanation for the heightened cardiovascular morbidity that is observed in patients with moderate-to-severe diseases.

Additionally, even after controlling the conventional risk factors like smoking, diabetes, and hyperlipidemia, moderate-to-severe psoriasis has been found to be an independent risk factor for significant adverse cardiovascular events, such as Myocardial Infarction and Stroke.^5^,^9^-^11^ These results highlight the necessity of proactive therapy and early cardiovascular risk assessment in psoriasis patients to avoid negative consequences.

The risk of cardiovascular morbidity and death, which is associated with severe psoriasis, can be considerably reduced by optimizing psoriasis treatment. Methotrexate has a cardioprotective effect, reducing the risk of acute myocardial infarction and cardiovascular events, according to research on psoriasis patients.^7^ The current study aimed to evaluate the frequency and associations of cardiovascular diseases in psoriasis patients, providing insights into the cardiovascular burden and emphasizing the importance of comprehensive care in this population and assess the importance of starting early immunomodulatory drugs.

## METHODOLOGY

The Section of Dermatology, outpatients’ department, at Aga Khan University Hospital (AKUH), Karachi, carried out this cross-sectional study between February 9, 2022, and August 8, 2022. The EpiInfo version 3.01 sample size calculator was used to determine the sample size that is 158.

### Ethical Approval:

Prior to sample collection an ethical approval from the IRB 2021-6208-18558 dated July 12, 2021 and informed consent was taken. Patients who had been diagnosed with clinical and/or biopsy-confirmed psoriasis were eligible to participate in the study, which employed non-probability consecutive sampling. The Severity of psoriasis was assessed by Psoriasis Area Severity Index (PASI).

A detailed medical history was obtained from all participants regarding previously diagnosed cardiovascular conditions, including structural or valvular heart diseases. Information was based on documented medical records and prior physician diagnoses. No routine echocardiographic screening was performed as part of the study protocol; therefore, only pre-existing or clinically recognized valvular abnormalities were recorded.

### Statistical Analysis:

To analyze the data, SPSS version 19.0 was used. The normality of continuous variables was evaluated using the Shapiro-Wilk test; a p-value of more than 0.05 is regarded as normal. Qualitative factors were stated as percentages and frequencies, whereas quantitative variables were presented as mean ± standard deviation.

## RESULTS

**Fig.1:**
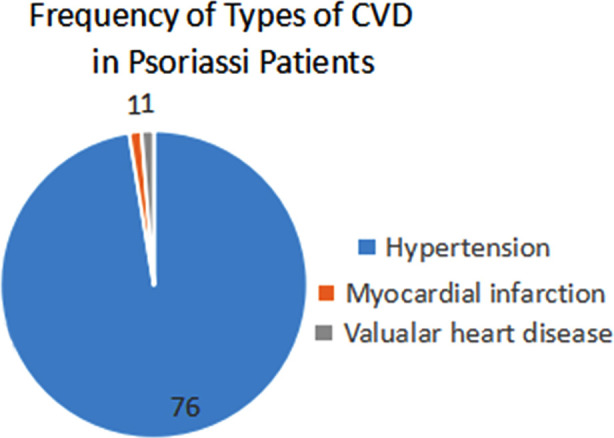
Frequency of types of Cardiovascular Diseases in Psoriasis patients.

The pie chart shows the frequency of CVDs in psoriasis patients, with hypertension being the most common, affecting 76 patients (48.1%). Myocardial infarction and Valvular Heart Disease follow with one, one case, is the least frequent. It also highlights the dominance of hypertension among the patients (Fig.1). The Shapiro-Wilk test (Table-I) was applied to assess the normality of variables, revealing significant deviations in age (41.98 ± 14.41 years, p = 0.0001), BMI (27.83 ± 5.15, p = 0.036), PASI score (5.87 ± 4.97, p = 0.0001), and disease duration (8.41 ± 7.17 years, p = 0.0001). A significant association was observed between PASI scores and the cardiovascular disease (CVD) (p = 0.029), as shown in (Table-II), with more severe cases showing a higher prevalence of CVD. Regarding systemic treatments, Acitretin was significantly associated with CVD (p = 0.044), whereas methotrexate (p = 0.941), cyclosporin (p = 0.939), and biologics (p = 0.314) did not exhibit significant associations. Stratified demographic analysis (Table-III) indicated a significantly higher prevalence of CVD in individuals older than 40 years (p = 0.0001). However, no significant association was found concerning gender (p = 0.997) or disease duration (p = 0.151) (Table-III). These findings underscore the importance of age, PASI severity and Acitretin use, as factors associated with CVD in psoriasis patients, while gender and disease duration were not significantly correlated.

**Table-I:** Descriptive Statistics of Shapiro-Wilk Test.

Variable	Mean±SD	P-value
Age	41.98±14.41	0.0001
Body Mass Index	27.83±5.15	0.036
PASI Score	5.87±4.97	0.0001
Duration of Disease	8.41±7.17	0.0001

Shapiro-Wilk Test was applied, level of significance <0.05.

**Table-II:** Association of Systemic Medicine with CVD in Psoriasis Patients.

			CVD		P value
			Yes	No	
PASI	Mild		39	46	0.170
	Moderate		14	22	
	Severe		21	9	
Systemic Medicine	Methotrexate	Yes	14	14	0.941
		No	64	66	
	Cyclosporin	Yes	15	15	0.939
		No	63	65	
	Acitretin	Yes	29	18	0.044
		No	49	62	
	Any other	Yes	28	35	0.314
		No	50	45	

Applied Chi-Square test, level of significance <0.05.

**Table-III:** Stratification of demographic factors with cardiovascular disease.

n=158		CVD		P-Value
		Yes	No	
Age Group	18 – 40 years	27 (17.1%)	54 (34.2%)	0.0001
	>40 years	51 (32.3%)	26 (16.5%)	
Gender	Male	38 (24.1%)	39 (24.7%)	0.997
	Female	40 (25.3%)	41 (25.9%)	
Duration	1 – 8 year	44 (27.8%)	54 (34.2%)	0.151
	>8 year	34 (21.5%)	26 (16.5%)	

Applied Chi-Square test, level of significance <0.05.

## DISCUSSION

Environmental factors, immune system activity, and genetic predisposition all have an impact on psoriasis, a chronic inflammatory skin condition.^10^ It has a major negative impact on patient’s quality of life and causes substantial financial, emotional, and physical hardships.^11^ Excessive dendritic cell activation drives the mechanism, which in turn triggers T-cells and causes the release of inflammatory cytokines such as interleukin-2, interferon, and tumor necrosis factor-alpha (TNF-a). Epidermal inflammation brought on by this series of immunological reactions can result in the formation of red, scaly plaques and, in certain situations, psoriatic arthritis (PSA).^12^

Characterized by cycles of remission and flare-ups, psoriasis affects 1-2% of individuals worldwide.^5^,^9^,^13^ The disease commonly emerges between the ages of 15 and 35, significantly impacting patient’s daily lives.^14^ Beyond its physical effects, psoriasis also contributes to psychological distress and financial strain due to ongoing treatment costs and frequent medical visits. The present study included participants aged 18 to 80 years, with a median age of 40 years. Previous studies have reported comparable mean ages, such as 34.8 ± 14.7 years, 45 ± 20.5 years, and 44.61 years.^15^,^16^

In terms of gender distribution, 48.7% of the study participants were male, while 51.3% were female. Similar gender proportions were observed in earlier studies by Khan GA et al. (58% male, 42% female), Edson-Heredia E et al. (47.3% male, 52.7% female), and Chiu TY et al. (58.1% male, 41.9% female).^15^,^17^,^18^

This study identified hypertension as the most common cardiovascular disease (CVD) among psoriasis patients, with 97.4% of those diagnosed with CVD suffering from hypertension. Myocardial infarction and valvular heart disease were each observed in 1.3% of cases, while no incidences of atrial fibrillation, heart failure, or thromboembolic events were noted.^19^

In contrast, research conducted in Lahore, Pakistan, reported an 11.6% prevalence of hypertension among psoriasis patients, along with 2.3% for myocardial infarction and 1% for heart failure.^15^ Another study found a 1.7% prevalence of atrial arrhythmia in psoriasis patients.^18^

With respect to the single case of valvular heart disease observed, a causal relationship with psoriasis cannot be established. The diagnosis was based on prior clinical history, and the underlying etiology may have been rheumatic, degenerative, or other non-psoriatic causes. Given the cross-sectional design and absence of detailed cardiac evaluation, these findings should be interpreted cautiously, and it cannot be concluded that valvular involvement was directly attributable to psoriasis-related inflammation.

Despite the well-established association between psoriasis and an increased risk of ischemic heart disease that was reported in previous epidemiological studies, the frequency of myocardial infarction in our cohort were comparatively low. Several factors may explain this observation. First, the relatively younger age distribution of our study population, with a median age of 40 years, may have limit the occurrence of overt coronary events. Second, the cross-sectional design and modest sample size may has reduced the ability to detect less frequent outcomes such as ischemic heart disease. Furthermore, cardiovascular diagnoses were based primarily on documented medical history rather than systematic cardiac screening investigations, which may have result in under-detection of subclinical or silent coronary artery disease. Therefore, the true burden of ischemic heart disease in psoriasis patients may be underestimate in the present study, and larger prospective studies with comprehensive cardiovascular evaluation is warranted to better defines this association.

These findings reinforce the strong association between Psoriasis and Cardiovascular diseases, particularly Hypertension. The current study results align with global research, highlighting an increased cardiovascular risk among individuals with Psoriasis. The study underscores the importance of early cardiovascular risk assessment and intervention in psoriasis patients to prevent long-term complications.

### Limitations:

There are few limitations in this study as this study is a single centered study, only assessed known cardiovascular events and did not screen for risk factors, as no investigations were performed. Additionally, patients were not followed up for subsequent cardiovascular events, limiting the ability to evaluate long-term risks.

## CONCLUSION

Psoriasis is associated with a high prevalence of cardiovascular comorbidities, likely due to shared inflammatory pathways. Hypertension was the most frequent cardiovascular condition observed in our study. These findings underscore the need for routine cardiovascular risk assessment in patients with psoriasis.

### Author’s Contribution:

**PJK:** Concept, Study Design, Data Collection, Manuscript Writing, literature search, data interpretation and responsible for the accuracy of the study.

**ST:** Study supervision, critical review, Data analysis.

**SM:** Manuscript Review, Data Analysis.

**SN:** Technical guidance, data collection.

All authors have read and the final version.
